# Differential expression of a *WRKY* gene between wild and cultivated soybeans correlates to seed size

**DOI:** 10.1093/jxb/erx147

**Published:** 2017-05-02

**Authors:** Yongzhe Gu, Wei Li, Hongwei Jiang, Yan Wang, Huihui Gao, Miao Liu, Qingshan Chen, Yongcai Lai, Chaoying He

**Affiliations:** 1 State Key Laboratory of Systematic and Evolutionary Botany, Institute of Botany, Chinese Academy of Sciences, Beijing, China; 2 University of Chinese Academy of Sciences, Beijing, China; 3 Crop Tillage and Cultivation Institute, Heilongjiang Academy of Agricultural Sciences, Harbin, Heilongjiang, China; 4 College of Agriculture, Northeast Agricultural University, Harbin, Heilongjiang, China

**Keywords:** CT-rich regulatory motif, domestication, expression variation, seed size, soybean, *WRKY* gene

## Abstract

Soybean (*Glycine max*) probably originated from the wild soybean (*Glycine soja*). *Glycine max* has a significantly larger seed size, but the underlying genomic changes are largely unknown. Candidate regulatory genes were preliminarily proposed by data co-localizing RNA sequencing with the quantitative loci (QTLs) for seed size. The soybean gene locus *SoyWRKY15a* and its orthologous genes from *G. max* (*GmWRKY15a*) and *G. soja* (*GsWRKY15a*) were analyzed in detail. The coding sequences were nearly identical between the two orthologs, but *GmWRKY15a* was significantly more highly expressed than *GsWRKY15a*. Four haplotypes (H1–H4) were found and they varied in the size of a CT-core microsatellite locus in the 5'-untranslated region of this gene. H1 (with six CT-repeats) was the only allelic version found in *G. max*, while H3 (with five CT-repeats) was the dominant *G. soja* allele. Differential expression of this gene in soybean pods was correlated with CT-repeat variation, and manipulation of the CT copy number altered the reporter gene expression, suggesting a regulatory role for the simple sequence repeats. Seed weight of wild soybeans harboring H1 was significantly greater than that of soybeans having haplotypes H2, H3, or H4, and seed weight was correlated with gene expression, suggesting the influence of *GsWRKY15a* in controlling seed size. However, the seed size might be refractory to increased *SoyWRKY15a* expression in cultivated soybeans. The evolutionary significance of *SoyWRKY15a* variation in soybean seed domestication is discussed.

## Teaser

*SoyWRKY15a* is associated with seed size in wild soybean, and the diverged expression of this gene due to a *cis*-motif variation in the 5'-untranslated region was one feature distinguishing wild soybeans from cultivated soybeans.

## Introduction

The cultivated soybean (*Glycine max*) is an economically important crop providing high levels of protein, oil, and other nutrients for humans worldwide. Cultivated soybean was domesticated from its wild relative (*Glycine soja*) in China 3000–5000 years ago (Hymowitz, 1970). A variety of morphological and physiological changes, such as seed size, seed hardness, pod dehiscence, growth determinacy, and flowering time, have occurred during domestication that distinguish soybean cultivars from their wild relatives (Liu *et al.*, 2007). These distinguishing traits are collectively called the ‘domestication syndrome’ of soybean, and they were developed by human selection (Tian *et al.*, 2010; Dong *et al.*, 2014; Sun *et al.*, 2015; Wang *et al.*, 2016). The genetic changes underlying phenotypic and physiological alterations during artificial selection have been called ‘domestication’ genes (Doebley *et al.*, 2006). Several methods have been used to study these genes in crops (Feltus, 2014). These include quantitative trait locus (QTL) mapping (Mauricio, 2001), and QTLs have advanced our understanding of crop domestication (Doebley *et al.*, 2006; Olsen and Wendel, 2013). In soybean, QTL mapping has enabled the isolation of selected genes that govern flowering time (Watanabe *et al.*, 2011; Xia *et al.*, 2012), seed hardness (Sun *et al.*, 2015), determinacy (Tian *et al.*, 2010; Ping *et al.*, 2014), and shattering (Funatsuki *et al.*, 2014). However, most of the genes underlying soybean domestication are poorly known due to the complexity of the soybean genome. Seed size is a major factor affecting yields, and appears to be a prime domesticated trait in soybeans (Song *et al.*, 2007; Shomura *et al.*, 2008). *Glycine soja* has small seeds, whereas *G. max* produces large seeds (Chen and Nelson, 2004). Approximately 200 QTLs affecting seed weight have been identified (www.soybase.org), suggesting that soybean seed size is controlled by multiple genetic loci. However, only one seed size regulatory gene, *GmGA20OX*, has been characterized and linked to the identified QTLs of seed weight 10–11 (Lu *et al.*, 2016). Several genes associated with seed size have also been identified, mainly using reverse genetic approaches. These include the subtilase gene *SBT1.1* in *Medicago truncatula* and *Pisum sativum* (D’Erfurth *et al.*, 2012), *BIG SEEDS1* (*BS1*) in *G. max* and *M. truncatula* (Ge *et al.*, 2016), two cytochrome P450 *KLUH* (*KLU*) homologs *GmCYP78A5* and *GmCYP78A72* in *G. max* (Wang *et al.*, 2015; Zhao *et al.*, 2016), and a cell wall invertase inhibitor gene *GmCIF1* in *G. max* (Tang *et al.*, 2017).

Publication of the soybean cultivar Williams 82 genome (Schmutz *et al.*, 2010) and high-throughput sequencing technologies provided an opportunity to track the evolutionary history of domesticated soybean, and to dissect the phenotypic diversification at the genome level. Genome comparison revealed that only ~0.31% of the nucleotide sequences differ between the genomes of wild and cultivated soybeans (Kim *et al.*, 2010). Genes that underwent large sequence changes and acceleration in the rate of nucleotide changes during domestication were identified (Lam *et al.*, 2010; Chung *et al.*, 2014; Li *et al.*, 2014; Zhou *et al.*, 2015). The importance of gene expression divergence in both biological function and phenotypic diversity during crop domestication has also been demonstrated in other crops (Cong *et al.*, 2008; Studer *et al.*, 2011; Lin *et al.*, 2012). Comparative transcriptome analyses through RNA sequencing (RNA-seq) efficiently identify differentially expressed genes/unigenes (DEGs) between cultivars and their wild relatives (Koenig *et al.*, 2013; Yoo and Wendel, 2014). Further combinations of high-throughput sequencing approaches including comparative RNA-seq with QTL mapping could help identify genes that underlie domestication-related traits (Olsen and Wendel, 2013).

Acreage and yields of soybean in north-east China account for 33% and 44%, respectively, of the national total (Liu and Herbert, 2002). Understanding the genomic variation underlying the divergence of *G. max* and *G. soja* might benefit soybean cultivation and genetic improvement. Variation in expression, resulting from genomic variation, plays an essential role in morphological variation and ultimately speciation (Carroll, 2008; Romero *et al.*, 2012). We therefore investigated soybeans from north-east China to find DEGs during pod/seed development between *G. max* and *G. soja* using RNA-seq. We then mapped these DEGs with QTLs associated with seed size to identify the candidate genes controlling soybean seed development. A soybean *WRKY15*-like gene (*SoyWRKY15a*) was particularly interesting since its orthologs, *GmWRKY15a* in *G. max* and *GsWRKY15a* in *G. soja*, were differentially expressed during pod development. Plant WRKY proteins are also involved in many biological processes, such as immune response, abiotic stress, and developmental processes (Rushton *et al.*, 2010), such as embryogenesis (Alexandrova and Conger, 2002; Lagacé and Matton, 2004) and seed development (Sun *et al.*, 2003; Luo *et al.*, 2005). We also found that *SoyWRKY15a* was associated with seed size variation in wild soybean and that the diverged expression dosage of this gene due to a CT-rich motif variation in the 5'-untranslated region (5'UTR) could distinguish wild soybeans from cultivated soybeans.

## Materials and methods

### Plant growth conditions and material collection

Soybean (*Glycine max*) cultivar Suinong14 (SN14), wild soybean (*G. soja*) ZYD00006, and 121 accessions, including 48 cultivars and 73 wild relatives, were studied (see Supplementary Table S1 at *JXB* online). The collection constituted wild accessions and staple cultivars from north-east China. To determine gene expression profiles during pod development and RNA-seq analyses, SN14 and ZYD00006 were grown in a mixture of soil (pindstrup, Denmark) and vermiculite at 24–27 °C with a 14 h light:10 h dark cycle in a greenhouse at the Institute of Botany, Chinese Academy of Sciences (Beijing, China). The seedlings were watered with tap water every 3 d. Unfertilized flower buds and flowers (onset of corolla presence) were collected shortly after the beginning of flowering. Because the flowers did not fully open in the greenhouse, the stage of pod (fertilized ovary) growing within the closed corolla was defined as F0. The developing pods at 1, 3, 5, 7, 10, and 15 d (designated F1–F15) after F0 were sampled. To collect the seeds and the pod wall, the pods were opened along the dorsal and ventral sutures. Samples were collected in the morning at around 09.00–10.00 h. For population analyses, 121 accessions were grown at the Minzhu experimental plot of Heilongjiang Academy of Agricultural Sciences (Harbin, China) during 2012–2015. The pods at F7 were collected for gene expression analysis in the population. The tissue samples were immediately put in liquid nitrogen, and then stored at −80 °C. Samples were separately collected from at least three seedlings of each accession to provide three biological replicates.

### Measurements of agronomic traits

One hundred-seed weight of dried mature seeds was used as the descriptor of seed size. Leaflet length was measured as the average length of three fully expanded terminal leaflets from the upper third of a plant at their longest point, and leaflet width was measured at the widest point at the full bloom stage. Plant height, node number, internode length, branch number, and pod number were measured at the full maturity stage. Three plants of each accession were measured.

### RNA-seq and identification of DEGs

Total RNAs of five tissues from SN14 and ZYD00006, namely unfertilized flower buds, flowers, and pods at different stages (F3, F5, and F7), were used for RNA-seq. Total RNA was isolated using the SV Total RNA Isolation System (Promega, USA). To generate a representative transcriptome of soybean reproductive organs, RNA from unfertilized flower buds, flowers, F3, F5, and F7 of ZYD00006 were mixed equally and sequenced (designated Z). For an equivalent comparison of SN14 and ZYD00006, RNA from flowers, F3, F5, and F7 were mixed equally to detect DEGs (samples from SN14 and ZYD00006 were respectively designed as SA and ZA). RNA-seq was carried out at the Beijing Genome Institute (BGI) (Shenzhen, China).

Three cDNA libraries (Z, ZA, and SA) were sequenced using Illumina HiSeq 2000. The sequencing strategy of library Z was PE90 (paired-end 90 bp), and the sequencing strategy of libraries ZA and SA was SE50 (single-end 50 bp). After clipping the adaptor sequences and removing the low-quality reads, RNA-seq data from Z were assembled using the Trinity assembly program (Grabherr *et al.*, 2011). The assembled unigenes were compared with Williams 82 transcript sequences (ver. 189 from http://www.phytozome.net/soybean) through Blastn (e-value=1E-5). Only the best hit targets with identity >90% were used to evaluate the corresponding relationship between unigenes and genomic transcripts. The short reads of libraries ZA and SA were mapped to reference sequences (Z) using SOAP2 (Li *et al.*, 2009), and mismatches of no more than two bases were allowed in the alignment. The absolute value of log2Ratio ≥1 and FDR (false discovery rate) ≤0.01 was used as the threshold to identify DEGs (Audic and Claverie, 1997; Benjamini and Yekutieli, 2001).

### Quantitative trait locus resources

QTLs of seed weight/volume were collected from Soybase (www.soybase.org), and the QTL-related genomic region was derived from the Williams 82 sequence (Glyma.Wm82.a1 and Glyma.Wm82.a2) in Soybase. The proportion of the genome covered by these QTL intervals and the QTL intervals corresponding to the regulatory DEGs were respectively estimated by the union of the involved QTL intervals in each case relative to the genome size of Williams 82 (referring to Glyma.Wm82.a2).

### Sequence isolation and analysis

Total RNA was reverse-transcribed into cDNA using the M-MLV cDNA synthesis kit (Invitrogen, USA). Genomic DNA was extracted from leaves using the Plant Genome Kit (Tiangen, China). The cDNAs and genomic DNAs of the genes of interest were amplified using gene-specific primers (Supplementary Table S2), and cloned into pEASY-blunt cloning vector (TransGen, China). At least six positive clones were sequenced for each gene to verify the sequences. The genomic PCR products from populations were directly sequenced. Sequencing was commercially performed at BGI (Beijing, China). The sequence was aligned using Clustal X v2.1 (Larkin *et al.* 2007) for haplotype analysis. The *cis*-motif in the 2300 bp region of *SoyWRKY15a* upstream of the translation initiation site from Williams 82 was predicted in PlantCARE (Lescot *et al.*, 2002). The genomic sequence of *SoyWRKY15a* from 302 resequenced soybean accessions including wild soybeans, landraces, and cultivars (Zhou *et al.*, 2015) was used for linkage disequilibrium analysis. Heterozygous alleles were treated as missing data. The squared correlation coefficient (*r*^2^) and *P*-value for linkage disequilibrium of CT variation and other polymorphisms [single nucleotide polymorphisms (SNPs) and insertions/deletions (indels)] were calculated by TASSEL 3.1.0 (Bradbury *et al.*, 2007).

### Phylogenetic analyses

Sequences were aligned using Clustal X v2.1 with default parameters (Larkin *et al.*, 2007). Alignments were optimized via manual adjustment, and partial sequences with poor alignment were excluded. Unrooted maximum likelihood (ML) trees were constructed using PhyML v3.1 under a Jones–Taylor–Thornton model with 100 bootstrap resamplings (Guindon *et al.* 2010).

### Quantitative reverse transcription–PCR (qRT–PCR)

Total RNA was treated with RNase-free DNase I (Promega, USA), and the first-strand cDNA was synthesized with oligo(dT)_18_ primers following the instructions of the M-MLV cDNA synthesis kit (Invitrogen, USA). qRT–PCR analysis of each gene was performed on an Mx3000P QPCR system (Stratagene, Germany) using SYBR Premix Ex Taq (TaKaRa, Japan) and gene-specific primers (Supplementary Table S2). The soybean *Actin* (Glyma18g52780) was used as the internal control (Hu *et al.*, 2009; Li *et al.*, 2012) to quantify the gene expression.

### Transient gene expression assay

To produce the LUC (luciferase) reporter gene constructs, ~1.0 kb fragments upstream of the putative translation initiation site of *SoyWRKY15a* were amplified from SN14 and ZYD00006, respectively, and the mutated fragments were generated by two rounds of PCR using specific mutation primer pairs (Supplementary Table S2). The fragment was respectively fused into a pUC-35sLUC vector (producing firefly luciferase) to generate the corresponding construct. Each obtained reporter plasmid was sequenced to verify the sequence, and then co-transformed with the *35S:GUS* (β-glucuronidase) internal control into F7 pods and leaves of SN14 and ZYD00006 by particle bombardment using a Biolistic PDS-1000/He system (Bio-Rad Laboratories, USA). A 15 μl aliquot of 50 mg ml^–1^ microparticles (1.0 μm, Bio-Rad) was mixed with 5.0 μg of plasmid mixture of each *SoyWRKY15a:LUC* made and *35S:GUS* (w/w 4/1), vortexed with 2.5 M CaCl_2_ and 0.1 M spermidine for 3 min, and then successively washed with 70% and 100% ethanol. The particle–DNA complex was re-suspended in 30 μl of 100% ethanol three times. The bombardment helium pressure value was 1100 psi, vacuum pressure was 26 mmHg, and the bombardment distance was 6 cm. Soybean organs, after bombardment, were kept in the dark at 24 °C for ~24 h. The soybean tissues were then ground in liquid nitrogen, and the resultant powder was resuspended in 1× cell culture lysis reagent (Promega, USA). The LUC and GUS activity was detected according to methods in previous work (Jiang *et al.*, 2016) with a luminescence kit using *LUC* assay substrate (Promega, USA) and 4-methylumbelliferyl β-d-glucuronide assay buffer (Jiang *et al.*, 2016). The relative reporter gene expression levels were calculated as the *LUC/GUS* ratios.

### Statistical analysis

Statistical analyses were performed using Microsoft Excel 2003 and R (v3.2.3). The significance of differences was determined using the two-tailed Student’s *t*-test. Pearson correlation coefficients were calculated among the traits and gene expression levels. All test differences at *P*≤0.05 were considered to be significant.

### Data deposition

The sequences reported in the article have been deposited in the databases of the National Center for Biotechnology Information (NCBI) under accession numbers KY120976–KY121100 (*SoyWRKY15* genes) and SRP093400 (RNA-seq reads).

## Results

### Evaluation of DEGs in reproductive tissues of *G. max* and *G. soja*

**Fig. 1.**
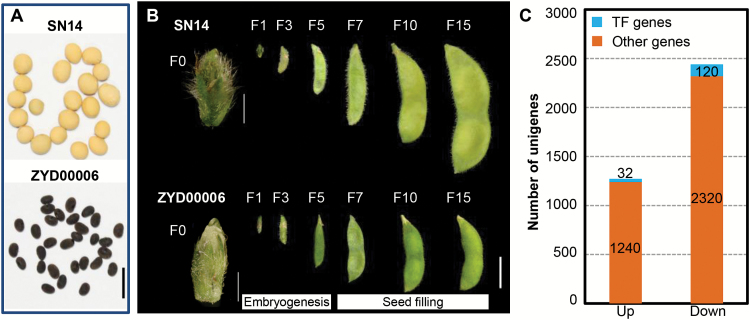
Morphology and development of soybean fruit and differentially expressed unigenes (DEGs) between SN14 and ZYD00006. (A) Mature seeds of SN14 and ZYD00006. Scale bar=1 cm. (B) Definition of pod development in SN14 and ZYD00006. F0 stage, onset of pod presence from the closed corolla; F1–F15, developing fruits 1–15 d after F0. Scale bars at the F0 stage are 1 mm and the scale bar for other developmental stages is 1 cm. According to a previous report (Le *et al.*, 2007), differentiation of embryo axis and cotyledons during embryogenesis occurs before the F3 stage, and predominance in cell expansion activity during seed filling starts after F5. (C) The number of DEGs during flower and pod development between SN14 and ZYD00006. The numbers in the column indicate the number of DEGs. TF, transcription factor. Up represents that gene expression in SN14 is higher than that of ZYD00006; otherwise it is indicated as Down.

Seed size is a major trait in domesticated soybean. Size contrast between cultivated and wild soybean is illustrated by cultivar Suinong 14 (SN14, 17.52 ± 0.54 g per 100 seeds) versus a wild soybean ZYD00006 (3.49 ± 0.10 g per 100 seeds) (Fig. 1A). To study genes related to soybean seed size, we identified the DEGs during pod development of cultivated and wild soybean. Pods of different developmental stages from SN14 and ZYD00006 were sampled. Young pods exiting the corolla were defined as F0. Developing pods at 1–15 d after F0 were designated F1–F15 (Fig. 1B). We sequenced the transcriptome of reproductive tissues including unfertilized flower buds, flowers, and developing fruits (stages F3, F5, and F7) of ZYD00006 (library Z), and used these as the reference sequence (Supplementary Table S3). Library Z contained ~125 274 unigenes with a mean length of 460 bp and an N50 length of 585 bp (Supplementary Fig. S1). These unigenes were aligned with the Williams 82 transcripts, and ~80.7% of the total unigenes were mapped on the soybean genome. The mapped unigenes were produced from 36 277 genes, so ~67.0% of the total soybean genes (54 175 genes in v189 models from Phytozome) were expressed during flower and pod development.

To detect DEGs, flowers and developing fruits (pods at stages of F3, F5, and F7) of SN14 and ZYD00006 (libraries SA and ZA, respectively) were collected for RNA-seq, and short reads from libraries ZA and SA were mapped against library Z (Supplementary Table S3). A total of 3712 unigenes, corresponding to 2462 genes (4.54% of the total genes), were differentially expressed among ZA and SA. Relative to ZA, 2440 unigenes were down-regulated while 1272 unigenes were up-regulated in SA (Fig. 1C). Among these, 152 unigenes encoding 125 transcription factors (TFs) were detected, and they belonged to 33 gene families (Supplementary Table S4; Supplementary Fig. S2). Given the important regulatory roles of TFs in plant development (Doebley and Lukens, 1998), we mainly focused on characterization of the DEGs coding TFs, also called regulatory DEGs.

### Screening candidate regulatory DEGs for seed size

To target candidate regulatory genes associated with seed size further, we used QTLs involving seed weight/size in soybean that were taken from Soybase (Supplementary Table S4). All these QTL intervals covered ~57.5% of genomes. We compared the chromosomal location of the identified regulatory DEGs and the detected QTLs affecting seed size (volume and weight). A total of 77 unigenes corresponding to 66 TF genes were located in the genomic regions on 17 chromosomes (occupying ~41.7% of the whole genome) that had the QTLs affecting seed size (Supplementary Table S4), suggesting the association of these genes with seed development.

**Fig. 2.**
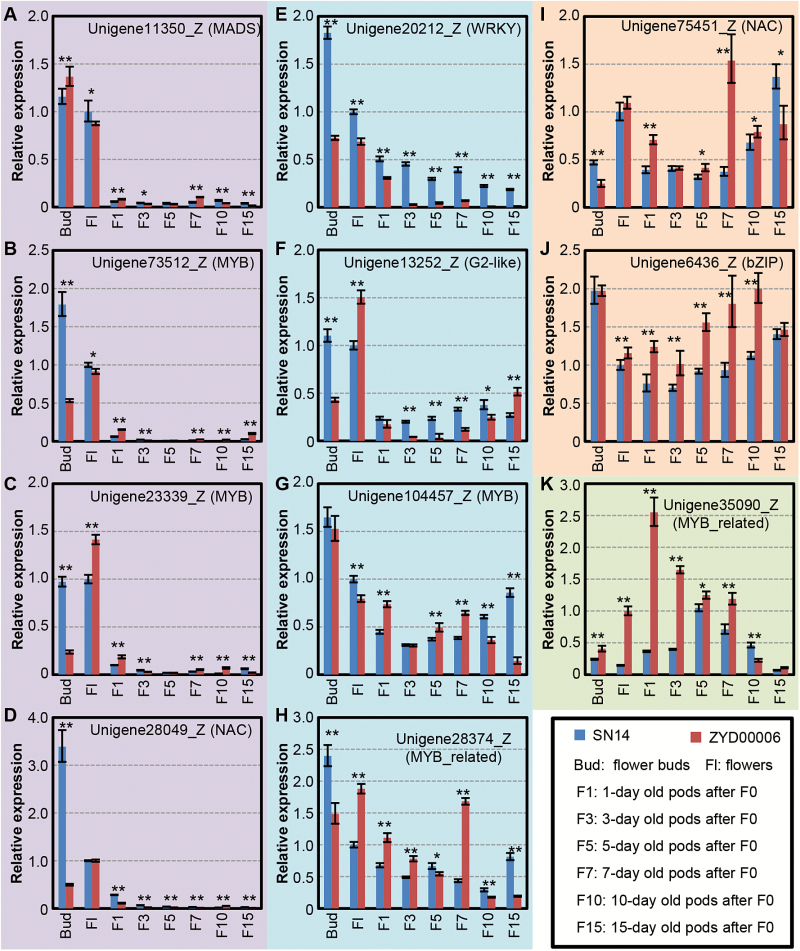
Expression of the candidate regulatory genes during pod development. (A) Unigene11350_Z. (B) Unigene73512_Z. (C) Unigene23339_Z. (D) Unigene28049_Z. (E) Unigene20212_Z. (F) Unigene13252_Z. (G) Unigene104457_Z. (H) Unigene28374_Z. (I) Unigene75451_Z. (J) Unigene6436_Z. (K) Unigene35090_Z. The transcription factor (TF) families are given in parentheses after the Unigenes. The unfertilized flower buds and different developing pod stages as indicated were harvested from *G. max* (SN14) and *G. soja* (ZYD00006). *Actin* (Glyma18g52780) is used as the internal control in qRT–PCR. The average relative expression and the SD are presented (*n*=3). Unigenes with a similar expression pattern are in the same background color. The blue column represents gene expression in SN14, while the red column indicates gene expression in ZYD00006. Asterisks indicate significance using Student’s *t*-test (*P<0.05; **P<0.01) when gene expression in SN14 was compared with that in ZYD00006.

To confirm this, we randomly selected 11 TF genes and studied gene expression profiles during seed development using qRT–PCR. Four types of expression profiles were observed during pod development (highlighted with different colors in Fig. 2). Four genes, including Unigene11350_Z, a MADS-box TF, were highly expressed in unfertilized buds and flowers and started to attenuate significantly at F1 (Fig. 2A–D). Four genes were highly expressed in the flower and early pod stage, and tended to decrease during pod development (Fig. 2E–H). Two genes were constitutively expressed during pod development (Fig. 2I, J). One gene was transiently expressed (Fig. 2K). On the one hand, the expression of all these genes was significantly different between SN14 and ZYD00006 at the F7 stage (Fig. 2), and at this stage cell expansion activity became predominant in soybean seed development (Fig. 1B), suggesting that it is crucial to check expression variation of these genes in the pods of the F7 stage for population analyses. On the other hand, these results were largely consistent with the differential expression pattern detected by RNA-seq analysis (Supplementary Table S5). The discrepancy between qRT–PCR and RNA-seq could be due to our pooling strategies in the two analyses. This involved pooling a mixture of selected soybean tissues for RNA-seq and a separate sampling of the corresponding materials of different developmental stages for qRT–PCR. Nonetheless, our results suggest potential roles for these genes in pod/seed development.

The average fold change of these genes was generally lower than that observed in RNA-seq (Supplementary Table S5). In particular, the expression of Unigene20212_Z in SN14 was greater than that in ZYD00006 during all stages, and showed the greatest difference among ZYD00006 and SN14 among the genes tested by qRT–PCR (Fig. 2E; Supplementary Table S5). Unigene20212_Z was a homolog of *AtWRKY15* (Supplementary Table S4) involved in cell expansion control in Arabidopsis (Vanderauwera *et al.*, 2012). This gene is located within the region of the four seed-weight QTLs on soybean chromosome 5 (Supplementary Fig. S3A). *AtWRKY44*, named *TRANSPARENT TESTA GLABRA* 2 (*TTG2*), and *AtWRKY10*, also called MINISEED 3 (MINI3), two members of group 1C of the WRKY family (Yin *et al.*, 2013; Supplementary Fig. S3B), were also demonstrated to control seed size in Arabidopsis (Garcia *et al.*, 2005; Luo *et al.*, 2005). These together indicate a role for a WRKY family in seed size control. Thus, our RNA-seq analysis provided useful, but preliminary, information. We therefore focused on characterizing Unigene20212_Z. A search of the William 82 genome revealed that Unigene20212_Z was the product of Glyma05g20710. Three additional close homologs were also present in the soybean genome. Phylogenetic analyses revealed that these four genes clustered into one clade containing *AtWRKY15* from Arabidopsis and *GhWRKY15* from cotton (Supplementary Fig. S3B) and belonged to group 2d of the WRKY family (Yin *et al.*, 2013; Supplementary Fig. S3B). Thus, these soybean homologs were all *SoyWRKY15* genes and were named *SoyWRKY15a* (Glyma05g20710), *SoyWRKY15b* (Glyma17g18480), *SoyWRKY15c* (Glyma01g39600), and *SoyWRKY15d* (Glyma11g05650).

### Expression profiles of *SoyWRKY15* genes during fruit and seed development

**Fig. 3.**
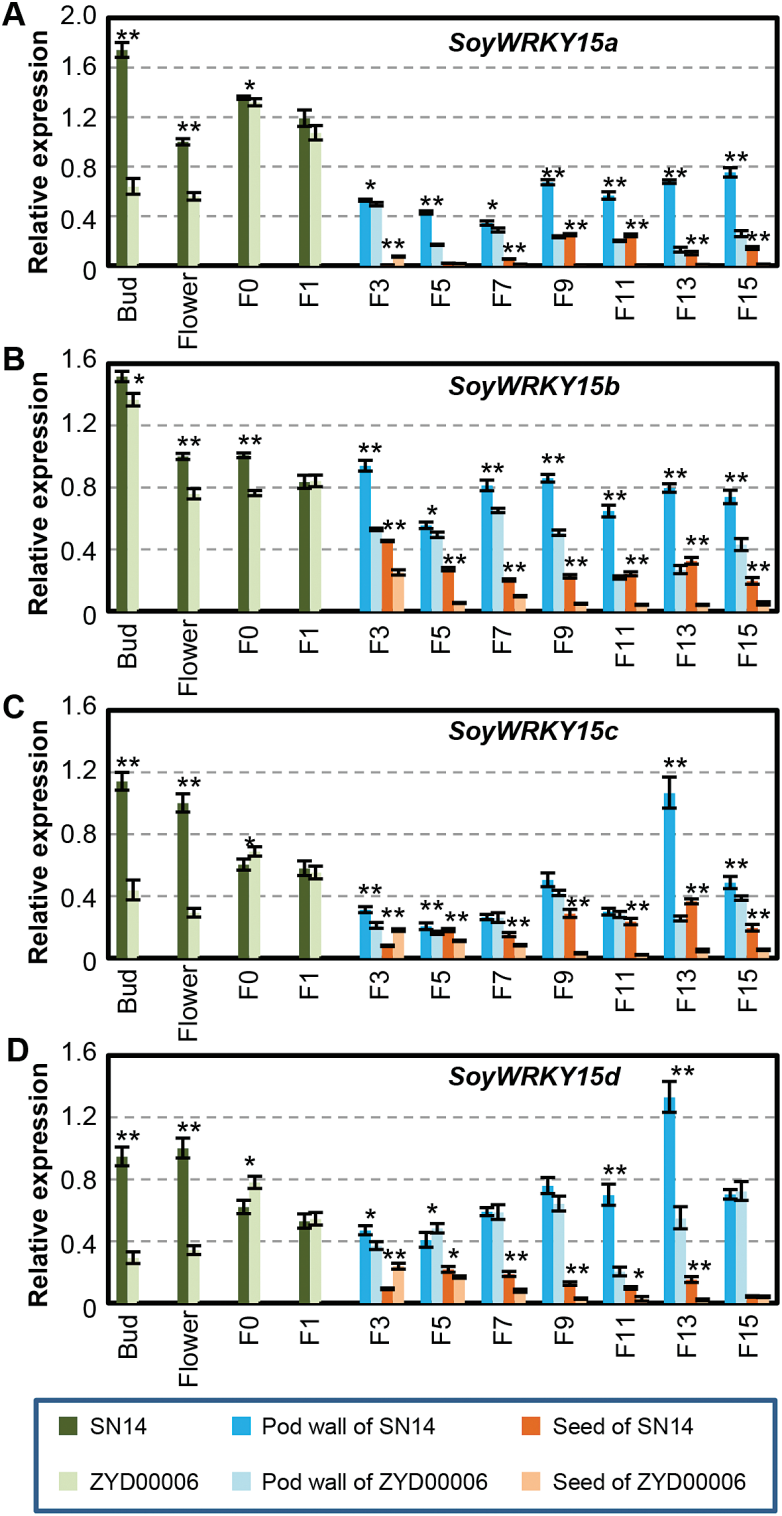
Expression of *SoyWRKY15* genes during fruit development. (A) *SoyWRKY15a.* (B) *SoyWRKY15b*. (C) *SoyWRKY15c*. (D) *SoyWRKY15d.* The tissues are unfertilized flower buds, flowers, and developing pods as indicated. The pods were divided into pod walls and seeds after the F3 stage. *Actin* (Glyma18g52780) is used as the internal control in qRT–PCR. The average relative expression and the SD are presented (*n*=3). The column legends of each graph are the same and are given underneath. Asterisks indicate significance using Student’s *t*-test (*P<0.05; **P<0.01) when gene expression in SN14 was compared with that in ZYD00006.

To determine the role of *SoyWRKY15* genes in pod/seed development, total RNAs of several developmental stages of flowers and fruits in soybeans were subjected to qRT–PCR analysis (Fig. 3). *SoyWRKY15a* expression during fruit development declined and the expression of *SoyWRKY15a* in the pod wall was higher than that in seeds (Fig. 3A). The maximum expression level of *GmWRKY15a* (the *SoyWRKY15a* gene in *G. max*) in SN14 occurred in unfertilized flower buds, while peak expression of *GsWRKY15a* (the *SoyWRKY15a* gene in *G. soja*) occurred in the F0 stage in ZYD00006 (Fig. 3A). However, the *GmWRKY15a* expression in SN14 was higher overall than that of *GsWRKY15a* in ZYD00006. Expression levels significantly diverged between the two accessions after the F7 stage, especially during seed development (Fig. 3A). *SoyWRKY15b*, *SoyWRKY15c*, and *SoyWRKY15d* generally had expression profiles similar to those of *SoyWRKY15a*, and these gradually decreased following pod/seed development. The expression of all these genes in SN14 was stronger than in ZYD00006 (Fig. 3), suggesting the roles of these genes in differential development of pod or seed size in SN14 and ZYD00006.

### *SoyWRKY15a* expression correlates to seed weight in soybean populations

**Fig. 4.**
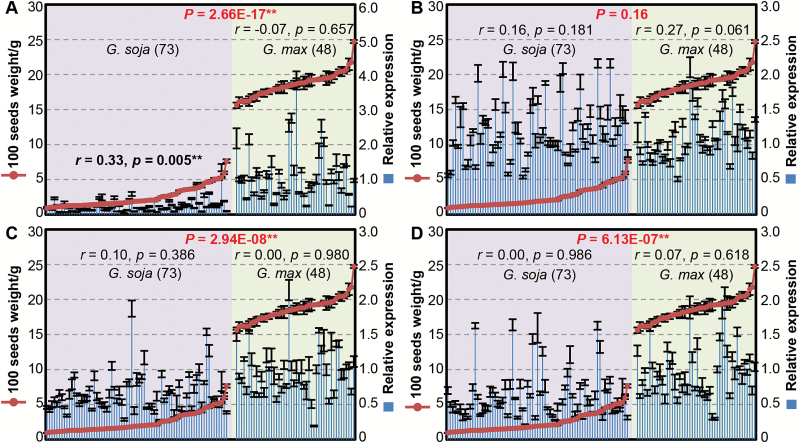
Correlation between *SoyWRKY15* gene expression and seed size. (A) *SoyWRKY15a*. (B) *SoyWRKY15b*. (C) *SoyWRKY15c*. (D) *SoyWRKY15d*. The expression level of each gene at the F7 stage was detected in 73 accessions of *G. soja* (pink background) and 48 accessions of *G. max* (green background). The mean relative expression and the SD are presented (*n*=3). The Pearson correlation coefficient of gene expression and 100-seed size (*r*) and *P*-value are given in each background. The *P*-value in red indicates the significance of the differential gene expression between wild and cultivated soybeans. All related information is presented in Supplementary Table S1.

To confirm this, the *SoyWRKY15* expression levels in F7 pods and 100-seed weight were studied in soybean populations consisting of 73 wild accessions and 48 cultivars. The two traits displayed significant variability among the populations (Fig. 4; Supplementary Table S4). The *SoyWRKY15a* expression levels were significantly different in wild and cultivated soybeans (*P*=2.66 × 10^17^; Fig. 4A), but no significant difference in *SoyWRKY15b* expression was observed between wild and cultivated soybeans (Fig. 4B). However, similar to *SoyWRKY15a*, the expression levels of *SoyWRKY15c* and *SoyWRKY15d* were also significantly different (Fig. 4C, D). We also performed correlation analysis between gene expression and seed weight. Only the expression level of *GsWRKY15a* was positively correlated with seed weight in wild soybean (*r*=0.33, *P*=0.005), but the *GmWRKY15a* expression level was not correlated with seed weight in cultivated soybean (Fig. 4A). No significant correlation between the *SoyWRKY15b*, *c*, and *d* expression level and seed weight was found in either wild or cultivated soybeans (Fig. 4B–D). These results suggest that expression of the *SoyWRKY15a* gene might influence soybean seed weight, particularly the seed/pod size variation in wild soybean, and thus may have promoted the divergence of *G. max* and *G. soja*.

### Evolutionary significance of *SoyWRKY15a* allelic variation

Sequence variation is often associated with functional divergence of the orthologous genes. We therefore investigated the allelic variation of *SoyWRKY15a* in both the coding sequence and the upstream putative regulatory sequence.

#### Conservation of the SoyWRKY15a coding sequence

Both cDNA and genomic DNA of *SoyWRKY15a* genes were isolated from SN14 and ZYD00006. Sequence comparison showed no variation in the exons and introns of *SoyWRKY15a* from the two accessions (Supplementary Fig. S4A). Unlike *SoyWRKY15a*, one insertion in the first exon (in SN14), one deletion in the second intron (in SN14), and two SNPs among SN14 and ZYD00006 were detected in the *SoyWRKY15b* locus (Supplementary Fig. S4B). Moreover, three non-synonymous mutations were observed (indicated with a red vertical line in Supplementary Fig. S4B). We also evaluated the sequence variation in the 302 re-sequenced accessions (Zhou *et al.*, 2015), and a synonymous SNP in the first exon was found in *SoyWRKY15a*, while five additional variations in *SoyWRKY15b* were detected (Supplementary Table S6). Seven and 16 variations, respectively, were found in *SoyWRKY15c* and *d* (Supplementary Table S6). These results suggest that *SoyWRKY15a* alleles are more highly conserved than other *SoyWRKY15* alleles, and suggest that the coding sequence variation of *SoyWRKY15a* was not involved in functional variation during the divergence of *G. max* and *G. soja*. Moreover, different alleles of the *SoyWRKY15a* gene, such as *GmWRKY15a* from *G. max* and *GsWRKY15a* from *G. soja*, might be conserved in function.

#### Upstream variation during allelic evolution of SoyWRKY15a genes

To account for the differential expression of the *SoyWRKY15a* genes in cultivated and wild soybeans, we compared the upstream putative regulatory sequences (UPRS), including the putative promoter and 5'UTR. The 2300 bp upstream fragments (from the predicted translation initiation site) from SN14 and ZYD00006 were subjected to sequencing analysis, and two variations were found. One was a 1 bp insertion at –716 in ZYD00006, and the other was a 2 bp deletion at –61 in the 5'UTR in ZYD00006 (Supplementary Fig. S5). The variation at –61 in the 5'UTR might lead to a change of the *cis*-motif identical to CTRMCAMV35S, an element in the *Cauliflower mosaic virus* 35S promoter (Pauli *et al.*, 2004). The difference in the CT-motif between SN14 and ZYD00006 may be related to differential gene expression. To explore further, we exploited the 302 re-sequenced soybean accessions (Zhou *et al.*, 2015). A total of 14 SNPs and five indels were detected in the 2300 bp UPRS. However, only the distributions of the SNP at –1880 and the indel at –61 were different between wild and cultivated soybeans (Supplementary Table S7). The SNP at –1880 was mainly related to the C/T transition and deletion (Supplementary Table S7). In wild soybean, C (45%) and T (40%) had approximately equal proportions, while T occupied ~76% and deletion accounted for ~17% in cultivated soybeans. However, the deletion at –61 in the 5'UTR was found in most of the wild soybeans (93.5%), but in only a small proportion (<8%) of landraces and improved cultivars (Supplementary Table S7). These data suggest that the CT-rich motif variation at indel –61 was involved in soybean domestication.

Besides the CT-motif variation, other polymorphic sites could also act as putative motifs to co-regulate *SoyWRKY15a* expression. To evaluate this, we performed association analysis. The 3.0 kb upstream sequences of the translation initiation site were investigated in the 302 soybean genome sequences (Zhou *et al.*, 2015). Due to the presence of only one homozygous allele (Supplementary Table S7), the association analysis was not conducted in wild soybeans. Three variations having high linkage disequilibrium with CT-motif variation (*r*^2^≥0.8), SNP_24874726 (the identified synonymous mutation at the first exon), SNP_24875569, and SNP_24875466, were observed in landraces but not in modern cultivars (Supplementary Fig. S6). Moreover, the major alleles of both SNP_24875569 and SNP_24875466 in the upstream non-coding region were consistent between wild and cultivated soybean (Supplementary Table S7), thus reducing the probability that these two variations were involved in soybean domestication. Therefore, the indel at –61 in the 5'UTR seemed to be a major and independent *cis*-motif variation, which may have contributed to the expression divergence of *SoyWRKY15a* between wild and cultivated soybean. In addition, some motifs are involved in stress and light responsiveness; *cis*-motifs required for endosperm expression were also found in *SoyWRKY15a* (Supplementary Table S8), consistent with the finding that *SoyWRKY15a* regulates seed development.

### Upstream sequence of *SoyWRKY15a* is associated with agronomic variation

**Fig. 5.**
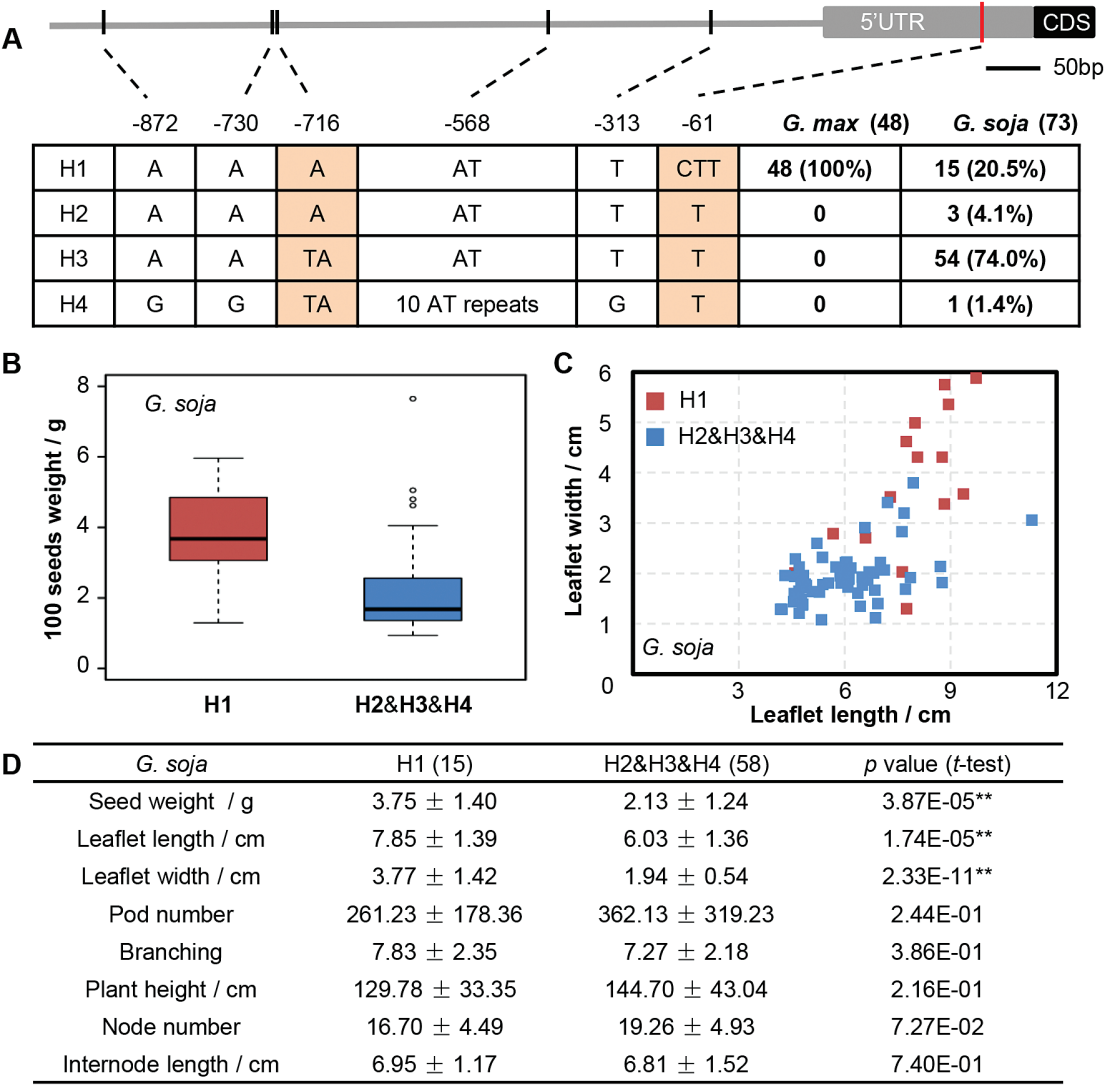
Association of the CT-motif variation in *SoyWRKY15a* and agronomic traits. (A) Nucleotide polymorphisms in the promoter and 5'UTR of the *SoyWRKY15a* gene. Black and gray boxes represent coding sequence and the UTR, respectively, and the horizontal gray line indicates the promoter region. The sites of variations are shown by vertical lines, and the deletion at –61 in UTRs is highlighted by a red vertical line. Four haplotypes (H1–H4) of the *SoyWRKY15a* gene were determined based on the polymorphisms detected in the investigated region. The polymorphisms that are different among H1 and H3 are shown on an orange background. The number and proportion of each haplotype in 48 cultivated and 73 wild accessions are given. (B–D) The effect of the defined *GsWRKY15a* haplotypes on seed weight (B), leaf size (C) and other agronomic traits (D) in wild soybean. The wild haplotypes (H2, H3, and H4) were considered together and compared with the domesticated H1. Mean ±SD is presented in (D). Significant differences (*P*-value) in the comparisons were detected using the two-tailed *t*-test.

To explore the influence of CT-motif variation on gene expression and plant morphology, the 1000 bp UPRS were isolated from 73 wild soybeans and 48 cultivars (Supplementary Table S4). Multiple sequence alignment revealed six polymorphic sites and defined four haplotypes, designed as H1, H2, H3, and H4 (Fig. 5A). All cultivated soybeans had H1 (Fig. 5A), suggesting that H1 might have been selected for during soybean domestication. Among the wild soybeans, 20.5% also had H1. Most wild accessions (74%) had H3, and fewer had H2 (4.1%) and H4 (1.4%) (Fig. 5A), and these haplotypes could be regarded as wild alleles. The distinguishing variations between H1 and these wild alleles were located to the CT-motif at indel –61.

**Fig. 6.**
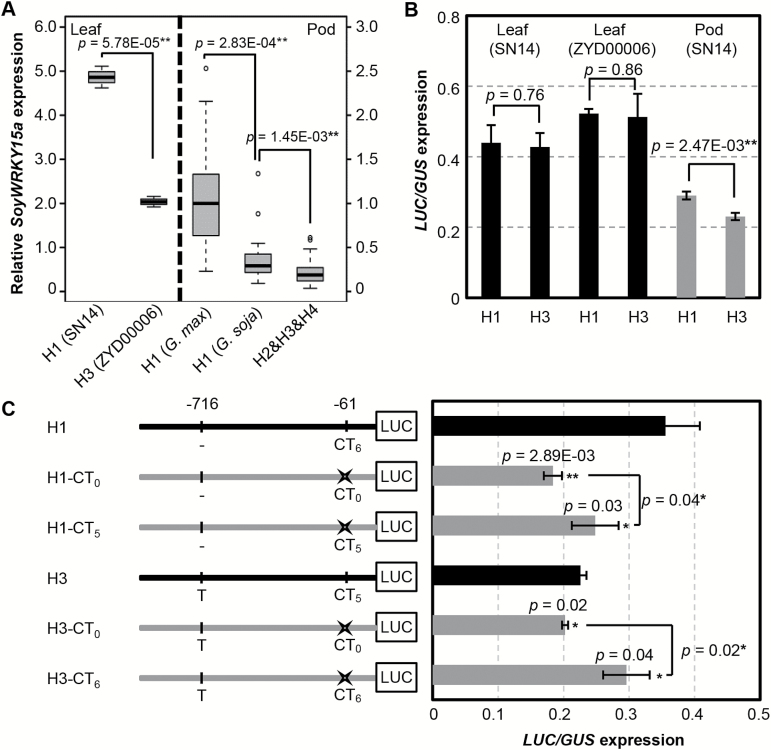
The CT-motif variation in *SoyWRKY15a* affects gene expression. (A) Expression of *SoyWRKY15a* with different types of CT-motif variation in leaves and F7 pods. The black dashed vertical line separates the leaves and pods as indicated. Left: the gene expression in leaves. H1 and H3 are from SN14 and ZYD00006, respectively. Right: the gene expression variation in pods. The wild haplotypes (H2, H3, and H4) were considered together and compared with the domesticated H1 in both wild and cultivated soybean. (B) Expression of the *LUC* gene driven by H1 from SN14 and H3 from ZYD00006. The black column indicates gene expression in leaves, and the gray column represents the gene expression in pods. (C) Diagrams of the reporter constructs and transient expression analysis. The mutated constructs harboring mutations in the CT-motif (in gray) in comparison with H1 and H3 of *SoyWRKY15a* (in black). CT_0_, CT_5_, and CT_6_ behind H1 and H3 indicate the number of CT-repeats, and CT_0_ is a result of complete deletion of the CT-motif. Relative expression of the *LUC* gene driven by these constructs is shown in the corresponding columns (*n* ≥3). The *P*-values of the two-tailed *t*-test are given in (A–C). In particular, the *P*-values in (C) were evaluated via comparison with each control (black column).

To explore further the role of the CT-motif variation of *SoyWRKY15a* related to morphological variation, we associated agronomic traits with haplotypes in wild soybeans. The 100-seed weight, leaf length, and leaf width of accessions with H1 were significantly greater than those of accessions with other haplotypes (Fig. 5B, C). However, pod number, branching, node number, internode length, and plant height were not associated with the UPRS variation of *GsWRKY15a* (Fig. 5D). H1 from SN14 was expressed significantly more highly than H3 of ZYD00006 in leaves, and H1 alleles were expressed at significantly higher levels than the other haplotypes even in wild soybean in pods (Fig. 6A). These results indicated that the CT-motif variation leads to differential expression of *SoyWRKY15a* and is further involved in controlling organ size variation, such as seeds and leaves, in wild soybean. However, the expression of *GsWRKY15a* H1 was significantly lower than that of *GmWRKY15a* H1 in pods (Fig. 6A), indicating that unidentified *cis*-elements or *trans*-acting factors may be involved in the regulation of *SoyWRKY15a* expression.

### CT-motif variation in the 5'UTR affects *SoyWRKY15a* expression in pods

*SoyWRKY15a* in leaves and pods of wild soybean was expressed at lower levels than in cultivated soybean (Fig. 6A). To characterize the regulatory role of the CT-motif functionally, we performed transient expression assays in these tissues. An ~1.0 kb UPRS from SN14 (H1) and ZYD00006 (H3) was fused to the *LUC* reporter gene and transformed into soybean leaves and pods of SN14 and ZYD00006, respectively. *LUC* expression was detected in both leaves and pods of SN14 but it was only detected in leaves of ZYD00006. Expression in the leaves of both species was comparable and stronger than expression in pods of SN14, irrespective of haplotypes (Fig. 6B). Consistent with the *SoyWRKY15a* expression variation among different haplotypes (Fig. 6A), the *LUC* expression in pods under H1 was significantly higher than that under H3 (*P*=0.00247) (Fig. 6B). However, the difference in *LUC* expression was not significant in leaves and did not reflect the *SoyWRKY15a* expression difference in leaves between SN14 and ZYD00006 (Fig. 6A, B). These observations suggest that these fragments were insufficient to drive an expression pattern identical to native expression of this gene in leaves, but it could characterize the expression variation in pods.

The *LUC* expression assay was unsuccessful in pods of ZYD00006 despite considerable effort, but the fragments H1 and H3 were able to drive *LUC* expression in pods of SN14. This indicated that differences in the pods between these two species can significantly affect manipulated gene expression and suggested that H1 and H3 can drive differential gene expression in pods. Two polymorphic sites (single nucleotide indel –716 and CT-motif variation at indel –61) were present in H1 and H3 (Figs 5A, 6C). Considering that the unique variation between H1 and the other haplotypes was related to indel –61, we manipulated the CT-motif and compared it with wild-type H1 (H1-CT_6_) and H3 (H3-CT_5_) (Fig. 6C). Deleting the CT-repeat (CT)_6_ for H1 and (CT)_5_ for H3, to give the constructs H1-CT_0_ and H3-CT_0_, and decreasing the copy number of the CT-repeats in H1 (H1-CT_5_) significantly attenuated *LUC* expression, whereas increasing the CT-repeat number in H3 (H3-CT_6_) enhanced the *LUC* expression level in pods (Fig. 6C). These results indicate that the CT-motif, as a *cis*-regulatory element in the UPRS, can influence the *SoyWRKY15a* expression in pods.

## Discussion

Cultivated soybean (*G. max*) and wild soybean (*G. soja*) share a common ancestor. Under domestication, a variety of agronomic traits of cultivated soybeans such as seed weight, seed hardness, and twinning habit are different from those of their wild relatives (Liu *et al.*, 2007). In soybean, a few domestication genes underlying morphological variation have been successfully characterized using QTL mapping (Tian *et al.*, 2010; Funatsuki *et al.*, 2014; Ping *et al.*, 2014; Sun *et al.*, 2015), but most mapped QTLs have not been cloned yet. In the present study, several possible genes involved in the divergence of *G. max* and *G. soja* were studied using DEGs in RNA-seq linked with the identified QTLs controlling seed size. The strategy of pooling tissues in different developmental stages in RNA-seq could mask genes whose expression is heterochronic between the two genotypes, but we found that differential expression of *SoyWRKY15a*, a member of the *WRKY* gene family, appears to be a good candidate for having played a role in soybean evolution, and is associated with seed size variation.

### *SoyWRKY15a* might regulate seed size in soybean

The WRKY family includes transcriptional regulators in plants (Eulgem *et al.*, 2000), that are involved in regulating plant immune responses and responses to abiotic stress (Eulgem and Somssich, 2007; Rushton *et al.*, 2010; Chen *et al.*, 2012). Members of this gene family are also involved in a variety of plant developmental processes (Rushton *et al.*, 2010), including senescence (Robatzek and Somssich, 2002) and trichome initiation (Johnson *et al.*, 2002). Overexpression of *AtWRKY15* results in increased cell expansion in Arabidopsis leaves (Vanderauwera *et al.*, 2012), and overexpression of *GhWRKY15* speeds up stem elongation in transgenic tobacco (Yu *et al.*, 2012). These results indicate that plant WRKY15 homologs can affect cell size and control organ size. Plant WRKY homologs also regulate embryogenesis (Alexandrova and Conger, 2002; Lagacé and Matton, 2004) and seed development (Sun *et al.*, 2003). Noticeably, the WRKY TF genes *TRANSPARENT TESTA GLABRA* 2 (*TTG2*) and *MINISEED 3* (*MINI3*) were functionally shown to regulate seed size (Garcia *et al.*, 2005; Luo *et al.*, 2005). Prevention of cell elongation in the integument in *ttg2* restricts endosperm and seed growth (Garcia *et al.*, 2005), while MINI3 binds to the *cytokinin oxidase* 2 (*CKX2*) promoter and activates *CKX2* expression to regulate endosperm growth (Li *et al.*, 2013). In the present study, we identified a small group of *WRKY* homologs from soybeans (*SoyWRKY15a*, *b*, *c*, and *d*) that were closely homologous to *AtWRKY15* and *GhWRKY15.* The four *SoyWRKY15* genes had similar expression profiles during pod/seed development in SN14 and ZYD00006. These results indicated that the *SoyWRKY15* genes have a common role in seed development. However, only *GsWRKY15a* expression correlated to seed size variation in wild soybeans. *SoyWRKY15a* was located near a previously identified seed-weight QTL cluster on chromosome 5 (Han *et al.*, 2012; Sun *et al.*, 2012). Therefore, *SoyWRKY15a* is a candidate gene for this QTL and may play a role in seed development and seed size control. Furthermore, *SoyWRKY15a* from the two soybean species shared a similar expression profile during seed development but they started to show significant expression divergence at the F7 stage, a stage at which cell expansion activity predominates, suggesting that *SoyWRKY15a* might be a cell size regulator. However, the coding sequence was identical, indicating that GmWRKY15a from *G. max* and GsWRKY15a from *G. soja* might have conserved biochemical and developmental roles.

Gene duplication and subsequent divergence can drive plant morphogenetic evolution (Rensing, 2014). The details of functional divergence of the four *SoyWRKY15* genes and the mechanism by which *SoyWRKY15a* regulates seed size require further study, but our work suggests that *SoyWRKY15a* is likely to be a cell size regulator and involved in seed size control. Moreover, *SoyWRKY15a* became most distinct among the four *SoyWRKY15* homologous genes and seems to be associated with soybean domestication.

### *SoyWRKY15a* variation pattern supports its role in soybean domestication

The distinguishing feature of the orthologous genes of *SoyWRKY15a* (*GmWRKY15a* and *GsWRKY15a*) is differential expression. In particular, *GmWRKY15a* expression was significantly higher than *GsWRKY15a* expression during pod development, indicating that this gene may play a role in domestic soybean traits. To better understand the gene expression divergence between wild and cultivated soybean, we compared the putative *cis*-regulatory motifs upstream of the *SoyWRKY15a* in *G. max* and *G. soja*. Four haplotypes (H1–H4) were defined in the 1.0 kb upstream fragment. All cultivated soybeans contained H1, while most wild soybeans were H3 (74%), demonstrating that H1 is the domesticated allele. The *GmWRKY15a* alleles were expressed at higher levels than the *GsWRKY15a* alleles, and *GsWRKY15a* H1 was also expressed at a level higher than other wild alleles. This suggests that a regulatory signal exists on H1 that enhances gene expression. The divergence between H1 and H3 involved an insertion at –716 and a deletion at –61. The insertion at –716 was in poly(T), whereas the deletion at –61 occurred in a CT-rich region, a small microsatellite element. The distinguishing sequence feature of wild and domesticated *SoyWRKY15a* alleles is the CT-core simple sequence repeat. Previous studies have demonstrated that increased CT number enhances gene expression (Xu and Goodridge, 1998; Pauli *et al.*, 2004; Yang *et al.*, 2013), and replacing CT copies by the same numbers of other nucleotides results in gene expression differences (Xu and Goodridge, 1998), indicating that the CT number is more important than spacing in gene expression regulation. In our transient assays, the *SoyWRKY15a* H1 and H3 haplotypes did not show differential expression in leaves, but expression was significantly different in pods, indicating that the *SoyWRKY15a* expression is dependent on *trans*-acting factors. This is supported by the finding that expression of the *GsWRKY15a* H1 haplotype was significantly lower than expression of the *GmWRKY15a* H1 haplotype *in planta*. The *trans*-acting factors involved in the expressional divergence of H1 in pods are unknown, but association analysis demonstrated that both gene expression (*GsWRKY15a*) and agronomic traits (seed size and leaf size) were significantly associated with the CT variation in wild soybean, indicating that CT-motif variation can influence gene expression. This theory was further supported by the finding that both H1 and H3 can drive differential expression of the reporter genes in pods and manipulation of the CT-repeat number in the two haplotypes can alter the expression of the reporter genes. The regulatory variation due to either CT-repeat number or spacing in this soybean CT-motif still needs further investigation; nonetheless, the CT-motif plays a regulatory role in *SoyWRKY15a* expression, and variation in the regulatory sequence and gene expression between wild and cultivated soybeans indicates that this gene was involved in soybean domestication.

Allelic variation of genes in the coding or regulatory regions can occur during crop domestication (Yamasaki *et al.*, 2005; Doebley *et al.*, 2006; Ross-Ibarra *et al.*, 2007). In our study, little coding variation in *SoyWRKY15a* was found, but the expression of *SoyWRKY15a* in wild soybean populations was positively correlated to seed size. Variable expression of this gene was also detected in a domesticated soybean population, but no correlation between *GmWRKY15a* expression and seed size was observed. This suggests that seed size may be refractory to increased *GmWRKY15a* expression in domesticated soybean. Therefore, expression of certain domesticated genes may correlate to phenotypic variation in wild populations but not in domesticated populations. This situation was also observed in the evolution of *GIa* (Wang *et al.*, 2016), an important domesticated gene controlling flowering time in soybean (Watanabe *et al.*, 2011; Wang *et al.*, 2016). Therefore, human selection of soybean traits might have favored mutated alleles controlling optimal/elite gene expression, while post-domestication selection focused on coding region variation of the selected genes or their related *trans*-acting regulators, ultimately resulting in modern cultivars.

The evolutionary roles of the proposed candidate orthologous gene pairs related to the divergence of *G. max* and *G. soja* need additional study. Nonetheless, we found that variation in expression of *SoyWRKY15a*, resulting from CT-copy variation of a microsatellite locus in the 5'UTR of this gene, might be involved in the regulation of seed size and may have been involved in soybean domestication. The CT-motif could be used as a functional marker in soybean breeding. This work provides new insights into genetic variation during soybean domestication and illustrates the essential role of differential gene expression in the evolution of plant morphology.

## Supplementary data

Supplementary data are available at *JXB* online.

Fig. S1. Length distribution of unigenes from library Z.

Fig. S2. Regulatory gene families that were differentially expressed.

Fig. S3. Identification of the *SoyWRKY15* gene family.

Fig. S4. Comparison of genomic structures of two *SoyWRKY15*-like coding regions.

Fig. S5. Upstream sequence alignment of *SoyWRKY15a* from SN14, ZYD00006, and Williams 82.

Fig. S6. Linkage analysis of the CT variation and adjacent polymorphic sites.

Table S1. Information on soybean accessions.

Table S2. Primers used in the study.

Table S3. General information about the RNA-seq libraries.

Table S4. Candidate regulatory DEGs and QTLs affecting seed weight/size.

Table S5. Candidate regulatory DEG expression between RNA-seq and qRT–PCR.

Table S6. Variations in the *SoyWRKY15* genes from the 302 resequenced accessions.

Table S7. Variations in the promoter and 5'UTR of *SoyWRKY15a* from the 302 resequenced accessions.

Table S8. Prediction of *cis*-elements in the promoter and 5'UTR of *SoyWRKY15a*.

## Author contributions

CYH and YZG conceived and designed the work. YCL and QSC were involved in work design; YZG conducted all experiments and helped with writing the manuscript; WL and ML performed field analysis. WL, HWJ, HHG, and YW participated in material preparation and gene expression analysis; YZG, CYH, YCL, and QSC analyzed the data; CYH wrote the paper. All authors read and approved the manuscript.

## Supplementary Material

supplementary_figures_S1_S6

supplementary_Tables_S1_S8

## Abbreviations:

QTL: quantitative trait locus
5'UTR: 5'-untranslated region
DEGs: differentially expressed genes
TF: transcription factor
qRT–PCR: quantitative reverse transcription–PCR.
