# Supplementary material for: Differential expression of a WRKY gene between wild and cultivated soybeans correlates to seed size
Source: J Exp Bot. 2017 May 2;68(11):2717–29. doi: 10.1093/jxb/erx147 (PMC5853923; doi:10.1093/jxb/erx147)
Supplement: supplementary_figures_S1_S6 [file erx147_suppl_supplementary_figures_s1_s6.pdf]

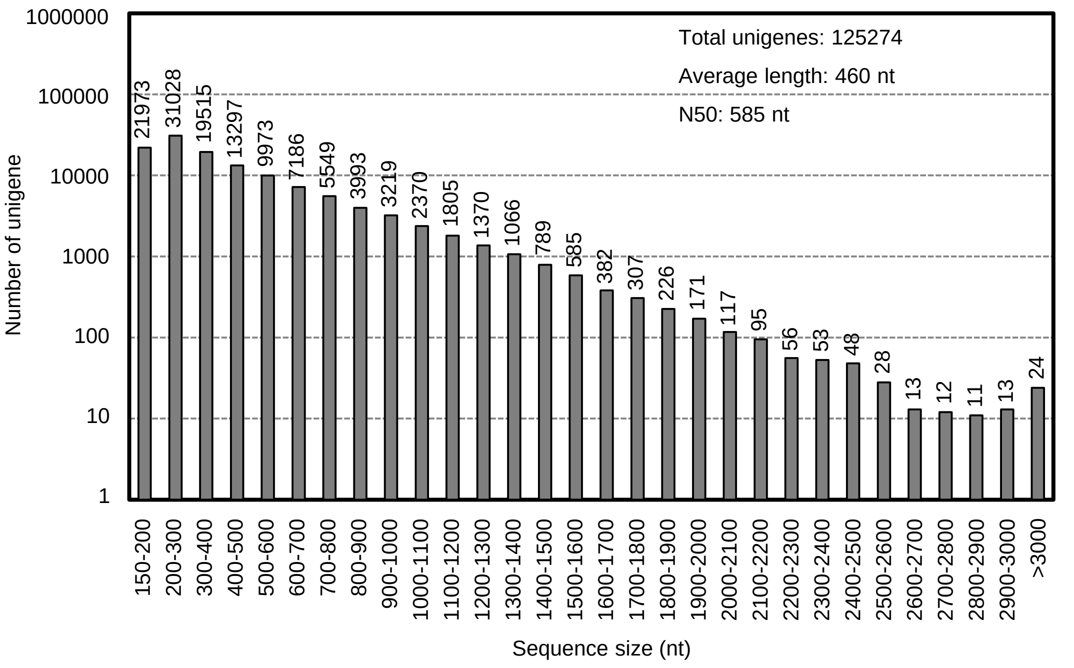

Figure S1 Length distribution of unigenes from library Z.
Library Z was constructed from a mixture of unfertilized flower buds, mature flowers and 3 stages of pods (F3, F5 and F7) of ZYD00006.

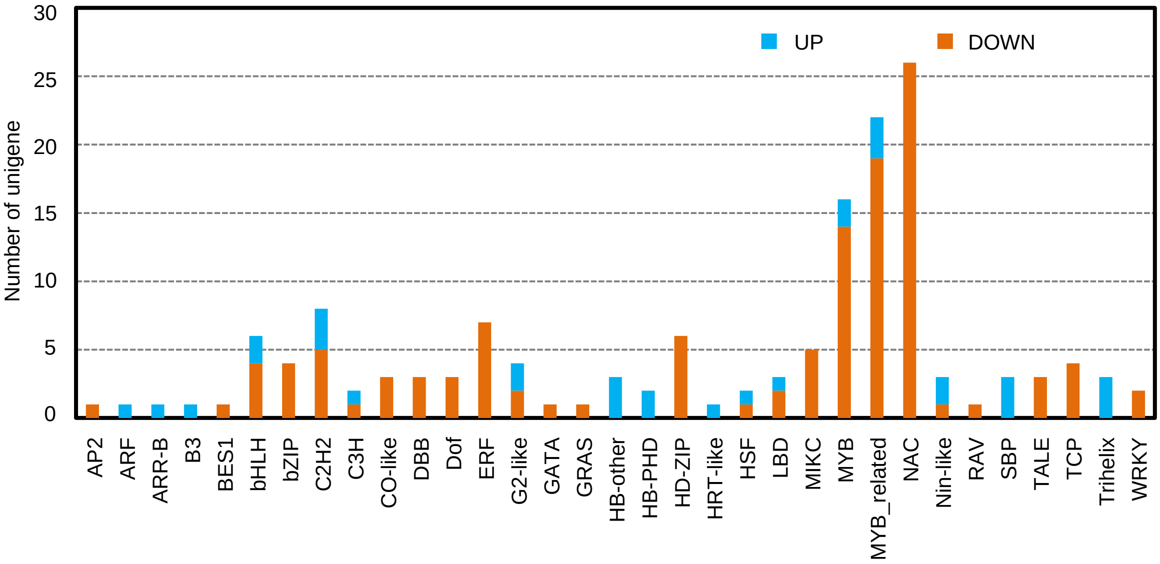

Figure S2 Regulatory gene families that were differentially expressed.
The UP represents that gene expression in SN14 is higher than that of ZYD00006, and the DOWN represents that gene expression in SN14 is lower than that of ZYD00006.

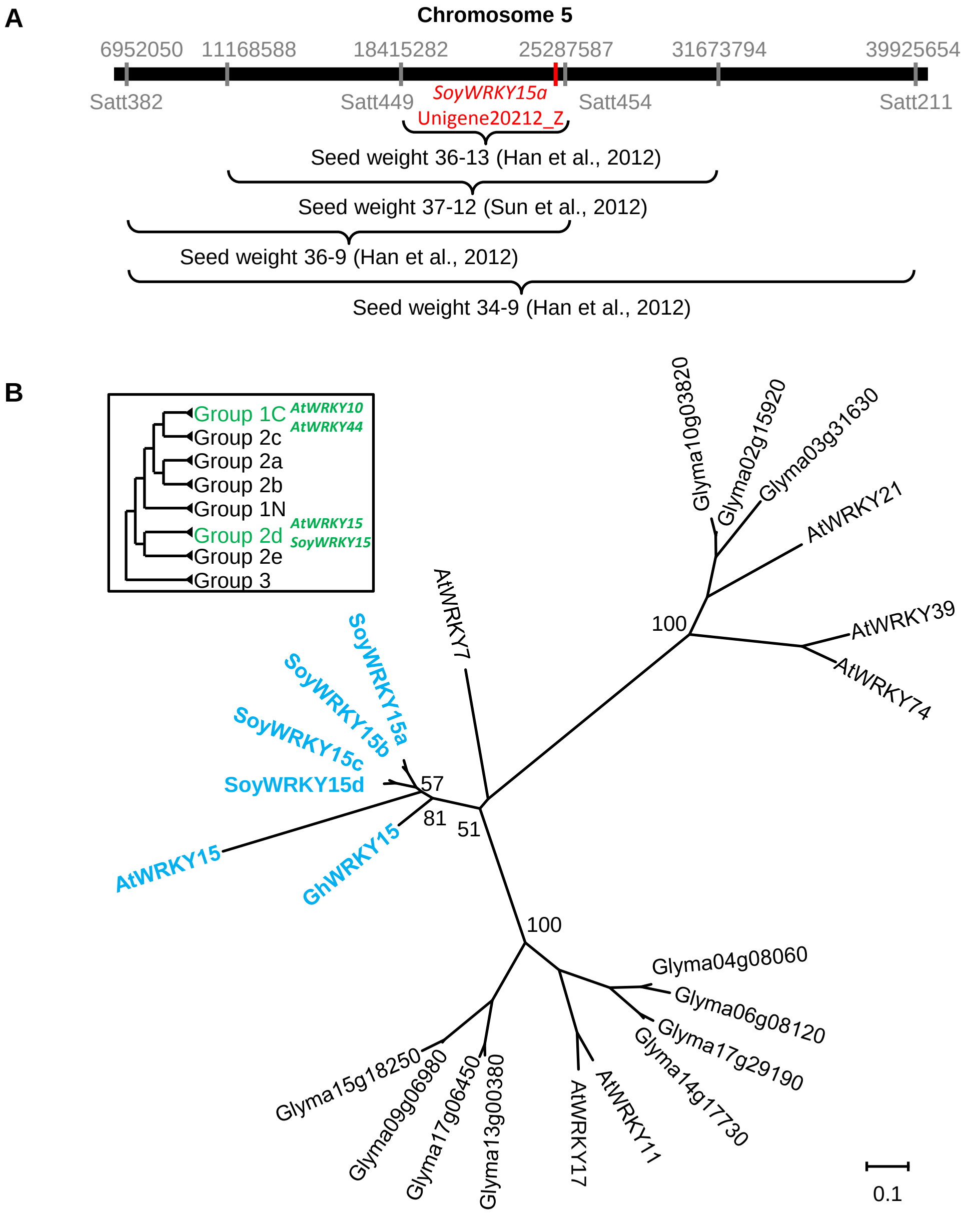

Figure S3 Identification of the *SoyWRKY15* gene family.
(**A**) Genomic region of *SoyWRKY15a* (Unigene20212_Z) and the related QTLs affecting seed size on chromosome 5. The backbone of chromosomal position referred to the Williams 82 sequence (Glyma.Wm82.a1) in Soybase. The red vertical line indicates the position of *SoyWRKY15a* (Unigene20212_Z). These QTLs as indicated were previously identified in multiple conditions (Han et al., 2012; Sun et al., 2012). (**B**) Phylogenetic analyses. A simple classification of the WRKY family in box was derived from the previously determined phylogenetic relationship (Yin *et al*., 2013), suggesting that *AtWRKY44* and *AtWRKY10* belonged to group 1C, and *AtWRKY15* and *SoyWRKY15* belonged to group 2d. Members of the two subfamilies highlighted in green were found to play roles in regulating organ size, such as seed and leaf. An unrooted maximum likelihood tree of group 2d were constructed, and the genes (accession number) included *SoyWRKY15a* (Glyma05g20710), *SoyWRKY15b* (Glyma17g18480), *SoyWRKY15c* (Glyma01g39600), *SoyWRKY15d* (Glyma11g05650), *AtWRKY21* (AT2G30590), *AtWRKY17* (AT2G24570), *AtWRKY15* (AT2G23320), *AtWRKY7* (AT4G24240), *AtWRKY11* (AT4G31550), *AtWRKY39* (AT3G04670), *AtWRKY74* (AT5G28650), GhWRKY15 (GU207869). The members belonging to the *WRKY15* family are highlighted in blue.

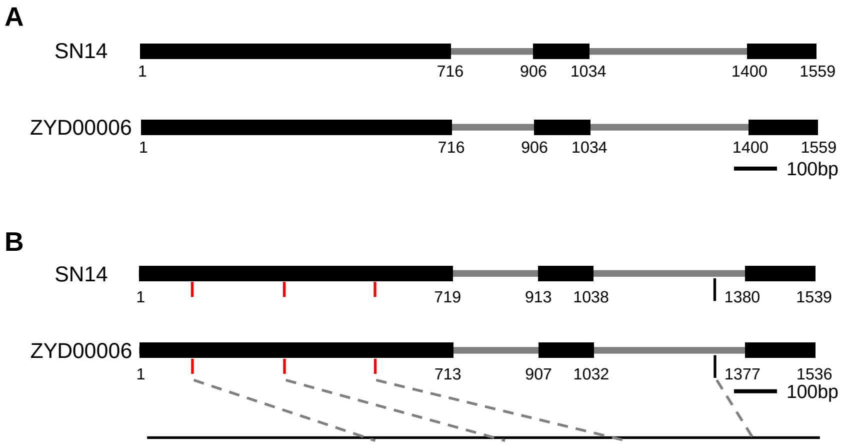

	162	338	481	1254
SN14	CCAAGCC	A	G	T
ZYD00006	C	T	T	TTGA

Figure S4 Comparison of genomic structures of 2 *SoyWRKY15*-like coding region.
(**A**) *SoyWRKY15a*. (**B**) *SoyWRKY15b*. The genomic sequence was isolated from SN14 and ZYD00006. Black boxes represent exons, and the connecting lines between the exons indicate the introns. Numbers below the gene structure represent the length from putative translation initiation site. No sequence variation in the defined region of *SoyWRKY15a* was observed between the 2 accessions, while the red vertical line and the corresponding number indicate the nonsynonymous mutations and position in the defined region of *SoyWRKY15b* between the 2 accessions.

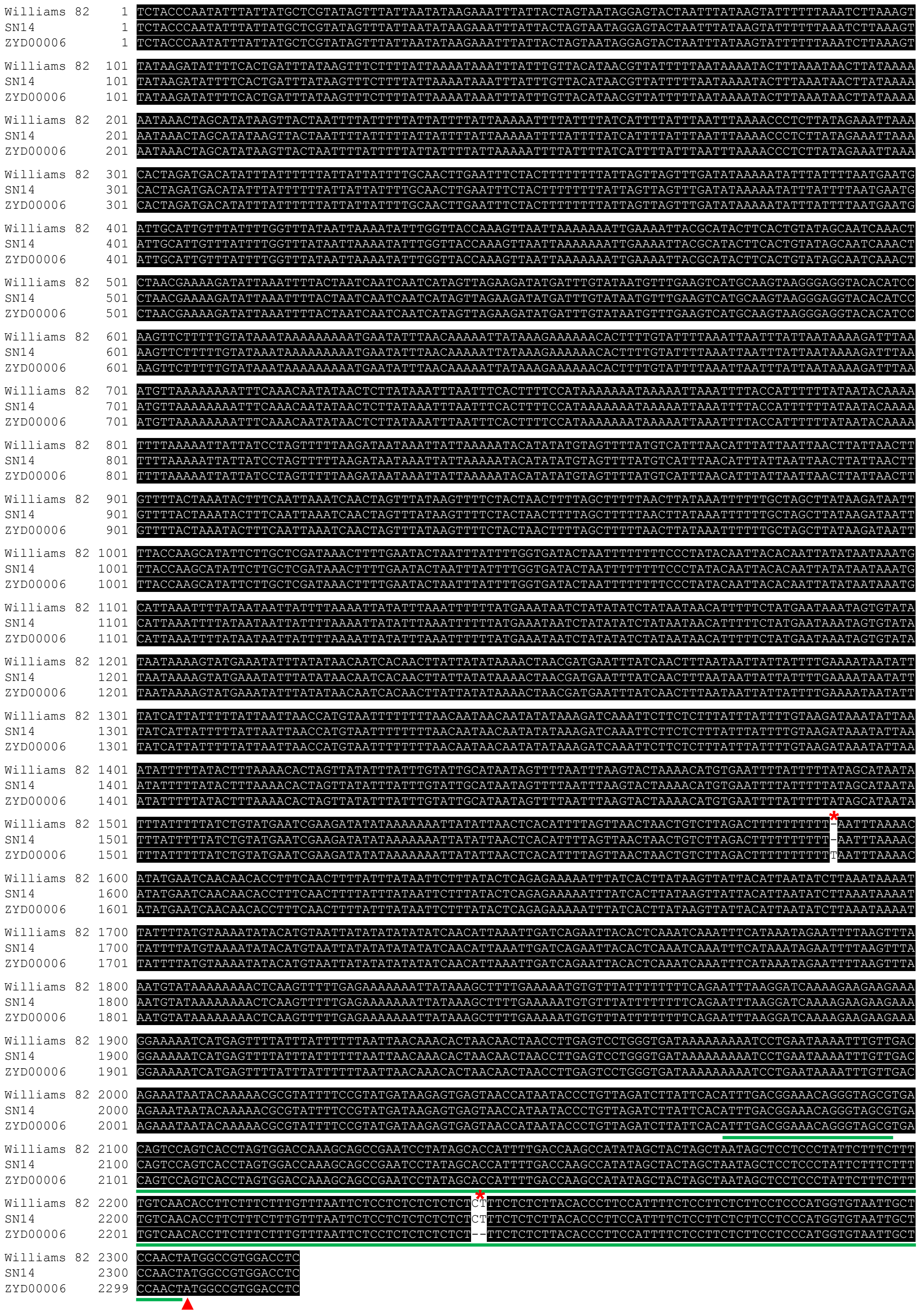

Figure S5 Upstream sequence alignment of *SoyWRKY15a* from SN14, ZYD00006 and Williams 82. The red asterisk indicates the position of variation. The red triangle indicates the predicted translation initiation site, and the predicted 5' UTR was underlined in green.

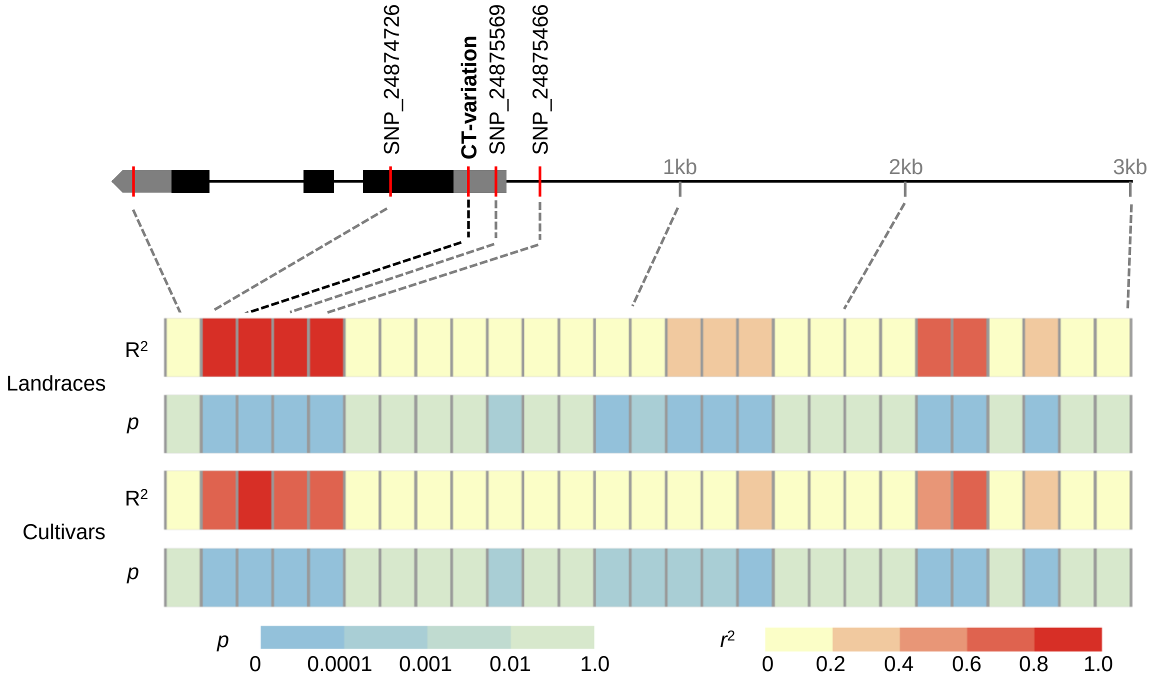

Figure S6 Linkage analysis of the CT-variation and adjacent polymorphic sites. The 3-kb upstream region of the *SoyWRKY15a* from the predicted translation initiation site was studied in landraces and cultivars. Black and gray boxes respectively represent exons and untranslated regions (UTRs), and the red line indicates the variation within the *SoyWRKY15a* locus. The correlation (r^2) and p value are given in each background as indicated.
